# Synergy of Artificial Intelligence and Laser Tech in Cosmetic Dermatology

**DOI:** 10.1111/jocd.16799

**Published:** 2025-03-15

**Authors:** Michael H. Gold, Mohamad Goldust

**Affiliations:** ^1^ Gold Skin Care Center Tennessee Clinical Research Center Nashville Tennessee USA; ^2^ Department of Dermatology Yale University School of Medicine New Haven Connecticut USA

**Keywords:** artificial intelligence, cosmetic dermatology, laser

Dear Editor,

This is to highlight the transformative advances at the connection of laser technology and artificial intelligence (AI) in cosmetic and aesthetic treatments. By integrating AI algorithms with laser devices, the fields of dermatology and plastic surgery are achieving huge levels of precision, efficiency, and safety, revolutionizing beauty enhancement and skin rejuvenation practices.

Traditionally, laser treatments relied heavily on practitioners' expertise to manually adjust parameters such as energy levels, pulse durations, and spot sizes according to individual patient characteristics. While this subjective approach was effective, it introduced variability and potential risk. The introduction of AI‐powered systems has redefined the standard of care. These systems analyze real‐time data and adapt parameters based on factors such as skin type, texture, pigmentation, and even environmental conditions. The result is consistently optimized outcomes with a reduced risk of complications [1, 2].

One prominent application of AI in dermatology is the use of AI‐guided fractional laser systems for skin resurfacing. These include non‐ablative fractional lasers, and ablative fractional lasers. These devices can utilize advanced AI algorithms, including convolutional neural networks for image analysis and deep learning models for real‐time optimization of treatment parameters. The algorithms analyze data from high‐resolution imaging systems to identify skin irregularities, such as fine lines, wrinkles, and scars. Based on this analysis, the devices can dynamically adjust settings like laser intensity, penetration depth, and pulse duration. This allows for precise targeting of the treatment areas while minimizing damage to the surrounding tissues. For example, deep learning models can predict the optimal energy delivery levels based on factors such as skin thickness and pigmentation, significantly enhancing both the safety and effectiveness of the procedures. This level of precision leads to better outcomes, shorter recovery times, and improved patient satisfaction [3, 4].

Another groundbreaking development can be AI‐powered hair removal devices, such as long‐pulsed Nd:YAG or alexandrite laser systems. Using AI‐driven real‐time monitoring, these systems can dynamically adapt laser energy to different hair densities and skin tones during a single session. This capability improves safety and efficacy, particularly for individuals with darker skin tones, who have historically faced a higher risk of adverse effects from traditional laser devices.

AI also plays a key role in predicting treatment responses. Devices like the KTP lasers can utilize machine learning algorithms to analyze historical patient data and identify patterns for tailoring treatment parameters for vascular and pigmented lesions. Through predictive analytics, clinicians can create more personalized treatment plans, increasing patient satisfaction and optimizing outcomes.

Additionally, AI‐based imaging platforms such as VISIA Skin Analysis seamlessly integrate with laser devices, providing detailed assessments of skin conditions, including UV damage, redness, and pigmentation irregularities. This data informs treatment protocols, ensuring a comprehensive and individualized approach.

AI‐driven automation facilitates processes such as patient assessment, treatment planning, and post‐treatment monitoring. For instance, hybrid fractional lasers (e.g., Sciton Halo) features an intuitive interface that guides practitioners through settings customized for each patient, significantly reducing procedure time while maintaining precision. This enhanced workflow efficiency allows clinicians to dedicate more time to consultations and education, ultimately improving the overall patient experience.

Despite these advancements, challenges remain. Ethical concerns related to data privacy, algorithmic bias, and the delegation of decision‐making to machines require careful consideration. Regulatory compliance and patient safety must remain at the forefront of these innovations. Collaboration among clinicians, engineers, and data scientists is essential for refining AI algorithms and effectively addressing these concerns [5].

The integration of AI with laser technology represents a huge change in cosmetic and aesthetic dermatology. The ability to deliver safer, more precise, and personalized treatments highlights the transformative potential of these innovations. As technologies such as deep learning and computer vision continue to evolve, we can expect further breakthroughs that redefine standards of beauty and self‐confidence.

The collaboration between AI and laser technology has started a new era in cosmetic dermatology, marked by improved outcomes, greater safety, and incomparable personalization. These advancements not only benefit patients but also enable practitioners to achieve exceptional results in the pursuit of beauty and skin health.

## Conflicts of Interest

The authors declare no conflicts of interest.

## Data Availability

Data sharing not applicable to this article as no datasets were generated or analysed during the current study.
